# Proceedings of the College of Physicians, of Philadelphia

**Published:** 1894-11

**Authors:** Lewis W. Steinbach


### Society Reports.

### PROCEEDINGS OF THE COLLEGE OF PHYSICIANS OF PHILADELPHIA.

### Section on General Surgery.

A stated meeting was held October 12, 1894, John B. Roberts, M. D., President, in the Chair.

### CASE OF GUNSHOT WOUND OF ABDOMEN AND LUNG.

By LEWIS W. STEINBACH, M. D.

A. H., aged thirty-six years, white, a Philadelphia police sergeant, was admitted to the Polyclinic Hospital, August 30, 1894. Twenty minutes previous to admission he had been accidentally shot in the abdomen by a 44-calibre pistol.

His temperature on admission was 98°, pulse 60, respiration 28. He was weak and faint, although externally he had not lost much blood. With the assistance of two officers he had walked from the place of shooting to the hospital, comprising several blocks.

Patient complained of some pain around umbilicus, and was unable to void his urine.

The history obtained from the patient states that he was sitting in a chair while the person who shot him was standing to his right, the pistol pointing slightly to patient's left. On examining the abdomen, a small wound one-quarter inch in diameter was seen two inches below and to the right of the umbilicus.

After cleansing the part a probe was gently inserted into the wound, and it was probed in all directions. The muscles had been torn up in several directions, so that this was not satisfactory, although there seemed to be a track in an upward direction and to the left, which from the history seemed to be the true course which the ball had taken. A small quantity of sterile water was then injected into the wound; but, as it returned, it could not be made out that the abdominal cavity had been entered, although this was what was thought to be the case.

Upon consultation with three of the hospital surgeons, it was decided to etherize the patient, follow the upward track, and if the abdominal cavity had been entered, to do a cœliotomy in order to ascertain the extent of injury.

Patient was etherized, and after all antiseptic precautions had been taken, a grooved director was introduced into the wound, and after some little trouble, as the track was irregular, it was laid open to about an inch in extent. Upon pushing the director further it entered the peritoneal cavity. The consensus of opinion was to do a laparotomy. A three-inch incision was made in the median line, and upon opening the peritoneal cavity a considerable quantity of blood escaped through the wound. The intestine was carefully examined, and nine perforations made by the bullet were found. These were principally in the lower part of the jejunum and the ileum. One was wholly in the mesentery, while the others chiefly lay at junction of it with intestine; and it was from these that the greater part of the blood was oozing. The colon was not perforated.

The various perforations were sutured with Lambert suture, silk being used. After carefully going over the small intestine, they were replaced and the abdominal cavity thoroughly washed with warm, sterile water until all blood and clots were removed, and the fluid returned clear. A glass drainage-tube was placed in the lower part of the wound, and silkworm-gut sutures introduced, closing the incision. The ordinary antiseptic dressing was applied.

The bullet had not been found, but was thought to have taken an upward course to the right of the spinal column.

The operation was a long one, and it was found necessary to administer strychnia and atropia to combat the shock. Temeratuie after operation was 99.8°; pulse, 120; respiration, 28.

The patient came out of the ether and seemed to rally; but, during the evening, his pulse became weak, thready, and very rapid, reaching 156 by 9 p. m. The temperature kept rising slowly and steadily until 3 a. m. it reached 102,4°. About ℥iv. fluid blood and serum was obtained through the drainage-tube. It did not clear, though it lessened in quantity toward morning. It was also noticed that the patient coughed up a small quantity of a dark chocolate-colored fluid. Stimulating treatment was kept up during the night. He complained greatly of thirst, was extremely restless, it being with difficulty he was restrained in bed. But at no time did he complain of pain.

The pulse became weaker and weaker, and at 8:36 o'clock the morning following operation, he died. Temperature taken half an hour previous to death registered 105.6°.

An autopsy was held by the coroner, and it was found that the bullet had pursued an upward course after striking the spinal column, passing beneath the diaphragm, rupturing some of the vessels at the root of the right lung, which was engorged with blood. The right pleura was filled with blood. There was also blood in the abdominal cavity, due to rupture of small vessels in liver tissue. The intestines looked ecchymotic in places; but the places that had been sutured showed commencing union. The bullet was not found, but traced to muscles of the back.

